# Postoperative Pain Following a Retroauricular Approach Versus a Transcanal Approach in Tympanoplasty Type 1: A 14-Day Retrospective Study

**DOI:** 10.3390/diagnostics16050675

**Published:** 2026-02-26

**Authors:** Wen-Ching Chuang, Li-Chun Hsieh, Chin-Kuo Chen

**Affiliations:** 1 Department of Otolaryngology-Head and Neck Surgery, Chang Gung Memorial Hospital, Taoyuan 33305, Taiwan; wenchingchuang0122@gmail.com; 2 Department of Otolaryngology Head and Neck Surgery, Mackay Memorial Hospital, Taipei 10449, Taiwan; lichunhsieh1978@gmail.com; 3 Department of Audiology and Speech Language Pathology, Mackay Medical University, New Taipei 25245, Taiwan; 4 Department of Medicine, Mackay Medical University, New Taipei 25245, Taiwan; 5 Department of Otolaryngology-Head and Neck Surgery, Chang Gung Memorial Hospital, Keelung 20401, Taiwan; 6 School of Traditional Chinese Medicine, College of Medicine, Chang Gung University, Taoyuan 33302, Taiwan

**Keywords:** tympanoplasty, postoperative pain, transcanal, retroauricular, analgesia, Wong–Baker FACES^®^

## Abstract

**Objectives**: This study aimed to determine whether the surgical approach used significantly affected postoperative pain and quality of life. **Methods**: This retrospective study included 45 adult patients undergoing type I tympanoplasty for chronic tympanic membrane perforation. The patients were divided into two groups: transcanal (n = 24) and retroauricular (n = 21). Postoperative pain was assessed using the Wong–Baker FACES^®^ Pain Rating Scale. Additional outcomes included analgesic use and activity limitation. **Results**: The graft success rates in the transcanal and retroauricular groups were 95.8% and 95.2%, respectively. The transcanal group reported significantly lower pain scores between postoperative days 5 and 8 (*p* < 0.05) and discontinued analgesic use earlier (mean 3.1 versus 4.3 days; *p* < 0.05). Furthermore, the transcanal group had fewer activity limitation events during recovery. Operative time was significantly shorter in the transcanal group (55.4 ± 10.1 versus 90.2 ± 10.6 min; *p* < 0.001). No major complications were observed in either group. **Conclusions**: A transcanal approach is associated with reduced postoperative pain, earlier recovery, and shorter analgesic use than a retroauricular approach in tympanoplasty type I.

## 1. Introduction

Postoperative pain is a common yet often underappreciated concern following otological surgery. Pain intensity in otologic procedures is generally considered mild to moderate [1,2]; however, transient discomfort can impede early recovery, delay hospital discharge, and negatively affect patient satisfaction and quality of life [3,4]. Effective pain control enhances patient experience, facilitates early mobilization, reduces the risk of postoperative complications, and lowers healthcare utilization [5,6].

Tympanoplasty, particularly type I procedures for tympanic membrane perforation repair, may be performed using either a retroauricular or transcanal approach. The transcanal approach offers advantages in postoperative wound care due to its minimally invasive technique. Additionally, the adoption of transcanal endoscopic ear surgery (TEES) has rapidly increased in recent years, providing superior surgical visualization and achieving graft success rates comparable to traditional retroauricular methods [7,8]. When tympanoplasty is performed using an exclusively transcanal approach, it eliminates the need for the extensive soft tissue dissection required when using a retroauricular incision. The transcanal approach is hypothesized to reduce tissue trauma, resulting in lower postoperative pain and faster recovery than a retroauricular approach. However, existing studies have yielded conflicting results. Baazil et al. reported no clinically meaningful differences in pain severity between endoscopic, endaural, and retroauricular approaches [4]. In contrast, Kwinter et al. observed that TEES in pediatric ossiculoplasty was associated with reduced postoperative pain compared to retroauricular techniques [9]. Notably, most prior studies have evaluated only short-term pain (within 7 days postoperatively) and did not address patient-reported quality of life over time.

The current study compares postoperative pain, recovery, and quality of life over a 14-day observation period between retroauricular and transcanal tympanoplasty. The hypothesis is that the minimally invasive transcanal approach is associated with lower postoperative pain and faster functional recovery than the traditional retroauricular technique. A 14-day observation window was chosen to capture the subacute resolution of postoperative discomfort and to reflect typical clinical recovery milestones.

## 2. Materials and Methods

### 2.1. Study Design and Ethical Approval

This retrospective comparative study was conducted in a tertiary referral medical center. This study was approved by the Institutional Review Board (IRB No.202500926B0) and conducted in accordance with the Declaration of Helsinki.

### 2.2. Patient Selection

Patients (aged 18–60 years) with simple tympanic membrane perforations were retrospectively reviewed. All patients were classified as American Society of Anesthesiologists physical status I or II and had no history of chronic pain or otologic surgery. The patients were divided into two groups based on the surgical approach and treatment period. The retroauricular approach group included patients who underwent tympanoplasty within 1 year prior to the introduction of endoscopic ear surgery at our institution. In contrast, the transcanal group comprised patients treated with tympanoplasty during the 2 years following the full implementation and routine adoption of standardized TEES. Patients with a history of ear surgery, ossicular chain defects, cholesteatoma, posterior canal wall overhang requiring canaloplasty, or unclear tympanomeatal annulus visibility were excluded.

### 2.3. Surgical Procedures

The patients were assigned to two groups based on the surgical approach and graft material used.

**Group I: Transcanal Tympanoplasty**

**Figure 1:**
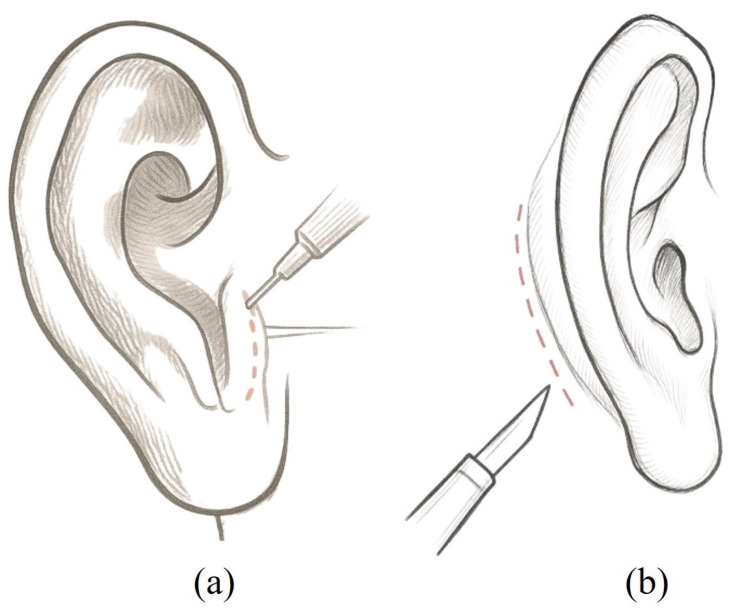
Comparison of external incisions between transcanal and retroauricular tympanoplasty. (**a**) Transcanal approach with tragal incision for graft harvesting. (**b**) Retroauricular approach.

Under general anesthesia, a U-shaped incision was made over the tragus to harvest the tragal perichondrium, with a margin approximately 1 mm larger than the tympanic membrane perforation. The perforation margins were denuded, and a 3 mm rigid endoscope (0° or 45°, Karl Storz) was used to elevate the tympanomeatal flap (Figure 1a). The harvested graft was dried, placed using the over-underlay technique, and positioned medial to the remnant tympanic membrane and lateral to the handle of the malleus. Gelfoam^®^ was applied to support the graft and pack the external auditory canal. Notably, there was no visible external scar following the transcanal surgery, highlighting the advantage of this minimally invasive approach that avoids a retroauricular incision.

**Group II: Retroauricular Tympanoplasty**

A retroauricular approach was used to harvest the temporalis fascia. Following the elevation of the tympanomeatal flap, the fascia graft was positioned using the over-underlay technique (Figure 1b). The flap was repositioned, and the external auditory canal was packed with Gelfoam^®^. The retroauricular incision was closed in layers with absorbable sutures for deep tissues and non-absorbable sutures for skin closure. A compressive ear dressing was applied postoperatively. Skin sutures were removed 1 week later during outpatient follow-up.

All procedures were performed by a single surgeon (C.-K. Chen), minimizing variability related to surgical technique or operator experience. All included cases were conducted after the surgeon had achieved proficiency in both microscopic and endoscopic tympanoplasty.

### 2.4. Otoscopy and Audiological Assessment

Otoscopic examination was performed bilaterally by an experienced otolaryngologist using a standard pneumatic otoscope. The purpose of the examination is to confirm the status of the external auditory canal and tympanic membrane, rule out abnormal conditions, such as foreign matter in the external auditory canal, cerumen blockage, external auditory canal inflammation, tympanic membrane perforation, and middle ear effusion, and evaluate wound recovery after ear surgery. If the above-mentioned lesions were found, the subjects were excluded from participating in the subsequent hearing examination and statistical analysis.

The hearing test was performed by an audiologist. The air conduction measurement frequency of the pure tone audiometry (PTA) covers 250 Hz to 8000 Hz, and the bone conduction measurement frequency covers 500 Hz to 4000 Hz. According to the World Health Organization (WHO) standard, hearing loss is defined as a PTA > 25 dB HL (average at 500 Hz, 1000 Hz, 2000 Hz, and 4000 Hz). The results of the hearing test can help diagnose the degree and type of hearing loss and decide whether hearing aids or other hearing rehabilitation are needed.

### 2.5. Postoperative Management and Pain Assessment

Postoperatively, all patients received a standardized regimen that included a short course of oral antibiotics and acetaminophen-based analgesics, as needed. Pain intensity was assessed daily using the Wong–Baker FACES^®^ Pain Rating Scale [10], a validated 0–10 visual analog scale administered consistently from postoperative day (POD) 1 to POD 14. The Wong–Baker FACES^®^ Pain Rating Scale (0–10) was used as the primary outcome measure for postoperative pain intensity. In addition, a six-point numerical scale embedded within the quality-of-life (QoL) questionnaire was used to assess patients’ overall well-being. The inclusion of these two instruments allowed differentiation between pain severity and its broader impact on daily functioning.

Patients completed a structured questionnaire assessing the following:

- **Pain-related interference with daily activities** was rated on a 6-point scale (0 = no limitation; 5 = extreme limitation);
- **Duration of analgesic use** was recorded in days;
- **Time to resume a normal diet** was recorded in days;
- **Overall satisfaction with the surgical experience** was rated on a 5-point Likert scale (1 = very dissatisfied; 5 = very satisfied).

A structured questionnaire was administered to assess postoperative quality of life, including pain intensity and activity limitation after surgery [11]. Participants rated their perceived pain level over the first 14 postoperative days using a six-point numerical scale ranging from 1 (“no pain at all”) to 6 (“extremely severe pain”).

### 2.6. Analgesic Protocol

Postoperative pain management followed a standardized protocol as follows. Oral acetaminophen (500 mg every 6 h as needed) was prescribed for all patients. If the patient-reported pain score exceeded 4 on the 10-point visual analog scale (VAS), an additional dose of ibuprofen (200 mg per tablet) was allowed as a rescue analgesic. No opioid medications were prescribed. The “days of analgesic use” were recorded based on patients’ self-reported medication logs during the 14-day postoperative follow-up period.

### 2.7. Sample Size Calculation

A priori power analysis was conducted using G*Power version 3.1 [12]. Based on previous studies indicating a moderate-to-large effect size (Cohen’s d = 0.8), a minimum of 19 patients per group was required to achieve a statistical power of 95% at a two-tailed alpha level of 0.05.

### 2.8. Statistical Analysis

All statistical analyses were performed using Statistical Package for the Social Sciences version 25.0 (IBM Corp., Armonk, NY, USA). Continuous variables were reported as means ± standard deviations and compared between groups using the Mann–Whitney U test. Categorical variables were analyzed using the chi-squared test. A two-tailed *p* < 0.05 was considered statistically significant.

## 3. Results

### 3.1. Patient Characteristics

Table 1 presents the demographic and baseline clinical characteristics of the two study groups. The mean age at surgery was 45.5 ± 11.3 years in Group I and 47.2 ± 11.6 years in Group II, with no statistically significant difference between groups (*p* = 0.439). The mean operative time was significantly shorter in Group I (55.4 ± 10.1 min) compared to Group II (90.2 ± 10.6 min; *p* < 0.001). In total, 45 patients were included in the analysis. Group I included 24 patients (10 male and 14 female), and Group II included 21 patients (seven male and 14 female) (*p* = 0.759). Similarly, no significant difference was observed in perforation location: central perforations occurred in 87.5% of patients in Group I and 85.7% in Group II, while marginal perforations occurred in 12.5% and 14.3% of patients, respectively (*p* = 1.000).

### 3.2. Hearing Outcomes

At the 6-month follow-up, Group I had a graft success rate of 95.8%, where Group II had a success rate of 95.2% (*p* = 1.000). The mean preoperative air–bone gap (ABG) was 19.1 ± 10.1 dB in Group I and 22.8 ± 8.8 dB in Group II (*p* = 0.161). The mean postoperative air–bone gap (ABG) was 10.5 ± 9.1 dB in Group I and 14.5 ± 9.6 dB in Group II (*p* = 0.144).

### 3.3. Postoperative Pain Scores

**Figure 2:**
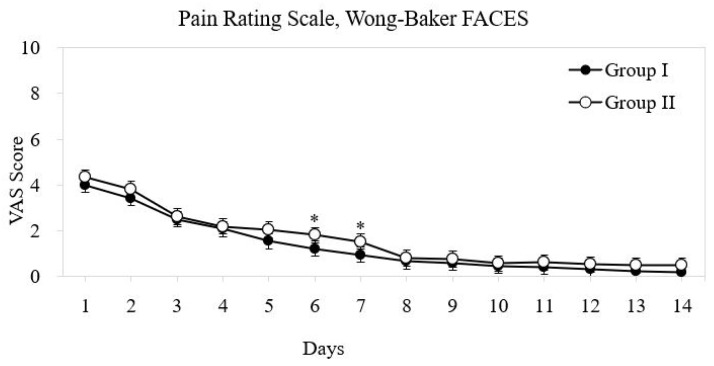
Daily postoperative pain scores (postoperative days [PODs] 1–14) comparing the transcanal and retroauricular groups. Mild = 1–3; moderate = 4–6; severe = 7–10.

Daily pain scores, as assessed using the Wong–Baker FACES^®^ Pain Rating Scale, declined over time in both groups. On POD 1, both groups reported moderate pain (mean scores 4–5). However, patients in Group I reported significantly lower pain scores on PODs 5 and 8 (*p* < 0.05) (Figure 2). The mean duration of analgesic use was also significantly shorter in Group I, with most patients discontinuing pain medication by POD 3 compared to POD 4 in Group II (*p* = 0.045).

### 3.4. Pain-Related Functional Limitation and Recovery

**Figure 3:**
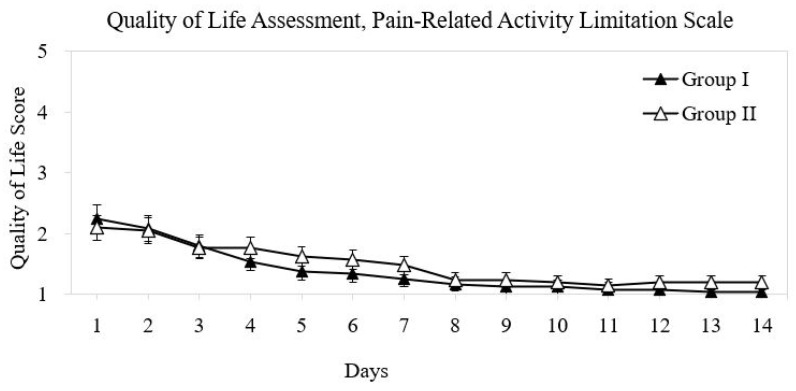
Daily pain-related activity limitation scores in both groups (POD 1–14). Score: 0 = no limitation; 5 = extreme limitation.

The assessment of pain-related interference with daily activities suggested faster recovery in Group I. By POD 3, most patients in Group I reported minimal or no functional limitations, whereas patients in Group II continued to report mild-to-moderate limitations until approximately POD 7 (Figure 3). However, this difference was not statistically significant.

Patient-reported satisfaction, measured using a 5-point Likert scale, was slightly higher in Group I (mean 4.4 ± 0.7) than in Group II (4.1 ± 0.7). However, this difference was not statistically significant (*p* = 0.237). Patients in Group I appeared to resume unrestricted daily activities earlier than those in Group II.

**Figure 4:**
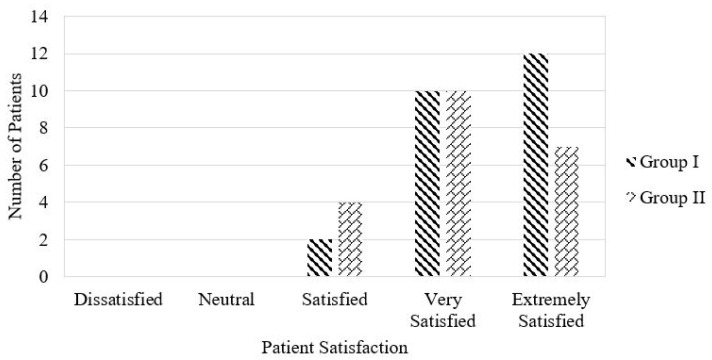
Distribution of patient-reported satisfaction.

The distribution of patient satisfaction is shown in Figure 4. None of the patients filled in dissatisfied or average; twelve patients in Group I and seven patients in Group II reported being extremely satisfied.

### 3.5. Adverse Events

No major postoperative complications were observed in either group. Minor complaints included a transient sore throat (related to endotracheal intubation) and temporary ear fullness. No cases of dizziness, sedation, or facial palsy were observed.

## 4. Discussion

This comparative study highlights the clinical advantages of a transcanal over retroauricular approach to tympanoplasty, particularly regarding postoperative pain control, surgical efficiency, and functional recovery over 14 days. These findings provide meaningful insights for otologic surgeons seeking patient-centered and minimally invasive approaches, particularly as outpatient tympanoplasty has become increasingly prevalent.

One of the most notable observations of this study was the shorter operative time in the transcanal group. This finding is consistent with previous studies reporting that the transcanal approach, which avoids a retroauricular incision and extensive tissue dissection, is associated with improved surgical efficiency. The proximity of the tragal perichondrium to the surgical field also facilitates graft harvesting, reducing operative exposure and potential donor site morbidity. In contrast, temporal fascia harvesting requires a separate surgical site and layered closure, which may contribute to longer operative duration and greater postoperative discomfort. Patients in the transcanal group generally reported lower pain scores from PODs 5 to 8, discontinued analgesic use earlier, and experienced fewer limitations during recovery. These findings suggest that the transcanal approach may be associated with less postoperative pain, particularly during the subacute recovery phase. The minimally invasive nature of transcanal tympanoplasty appears to support earlier resolution of discomfort, shorter duration of analgesic use (mean discontinuation on POD 3), and higher patient satisfaction, which together may contribute to more efficient outpatient recovery and healthcare resource utilization.

Postoperative pain is a critical determinant of surgical recovery and can influence patient mobility, satisfaction, and length of hospital stay. In otologic surgery, pain is generally considered mild; however, subtle differences in surgical techniques and tissue manipulation may contribute to variations in patient-reported outcomes. The lower pain burden observed in the transcanal group is consistent with the findings by Kwinter et al., who reported reduced postoperative pain and analgesic use in pediatric patients undergoing transcanal endoscopic ossiculoplasty compared to microscopic surgery [9]. Although pediatric results may not be directly generalizable to adults, our findings suggest a similar trend in adult tympanoplasty, underscoring the potential benefits of minimally invasive, endoscopic techniques in reducing patient discomfort. Furthermore, lower postoperative pain scores in the transcanal surgery group were consistent with those reported by Wang et al. Additionally, the self-rated appearance satisfaction of patients who underwent transcanal surgery was significantly higher than that of patients who underwent retroauricular surgery [13].

The observed differences between the two approaches likely reflect both the invasiveness of the surgical approach and the anatomical characteristics of graft harvesting. The retroauricular incision and temporalis fascia harvest activate branches of the great auricular, lesser occipital, and auriculotemporal nerves, causing sustained nociceptive signaling and subacute discomfort [14]. In contrast, tragal perichondrium harvest for transcanal tympanoplasty involves minimal and superficial tissue dissection, which reduces nerve stimulation and accelerates pain resolution [1,8].

These findings are further supported by broader evidence from the head and neck and neurosurgical literature. Li et al. demonstrated that surgical procedures involving posterior cervical or infratentorial dissection led to significantly higher pain scores within 72 h postoperatively, likely due to dense innervation and muscular overlap in those areas [15]. Analogously, the superficial and localized nature of the tragal graft harvest may contribute to the milder pain trajectory observed following the transcanal approach.

Additionally, operative time differed significantly between the two groups. Patients in the transcanal group underwent shorter surgeries (mean 55 versus 90 min), which may have contributed to differences in recovery. Extended operative time has been associated with greater tissue drying, prolonged retraction, and extended anesthetic exposure, all of which contribute to heightened postoperative pain and delayed wound healing [6,16]. Surgical duration itself is not a direct source of pain; however, it serves as a surrogate marker for procedural invasiveness and overall tissue trauma.

Despite overall favorable outcomes in both groups, only the transcanal group reached “no pain” status before POD 10. This finding may have practical implications for day surgery and enhanced recovery after surgery (ERAS) protocols. Rapid pain resolution, early cessation of analgesics, and quicker return to normal function align well with ERAS principles, which emphasize minimal analgesic use and early mobilization [17]. The transcanal approach to ear surgery appears to be compatible with these goals for more efficient outpatient recovery compared with conventional microscopic tympanoplasty.

Nevertheless, this study had some limitations. Its retrospective, time-separated design and non-randomized group allocation may introduce selection bias. However, the demographic and operative parameters were similar between the groups. The influence of graft donor site (temporalis fascia for the retroauricular group versus tragal perichondrium for the transcanal group) on postoperative pain is recognized as a possible confounder and should be considered in future prospective studies. Pain was assessed using the Wong–Baker FACES^®^ scale due to its accessibility, simplicity, and cultural familiarity of the scale support its use in our cohort. However, this scale is subject to individual interpretation and lacks granularity compared to numeric rating scales. Another limitation of this study is the temporal separation between the two study groups. Because the patients in the TEES group and the microscopic group were enrolled during different time periods, a potential learning-curve effect associated with the introduction of TEES might have influenced the operative time. However, the 14-day postoperative follow-up period provides a more reliable evaluation of patient-reported pain responses, which are considered genuine and less likely to be affected by surgical proficiency. Furthermore, the sample size was adequate for detecting primary outcome differences and limited subgroup analyses, such as sex differences and perforation characteristics. Future prospective randomized trials with larger cohorts and extended follow-up periods are warranted to validate these findings and explore their implications for long-term success and audiological outcomes.

In summary, this study provides several novel contributions compared with previously published literature. First, it extends the observation period to 14 days postoperatively, allowing assessment of subacute pain resolution and functional recovery beyond the early postoperative phase commonly limited to seven days in prior reports. Second, it focuses exclusively on adult patients undergoing type I tympanoplasty, ensuring a homogeneous surgical population and reducing confounding factors related to age and procedure type. Third, it offers a direct comparison between the transcanal and retroauricular approaches under standardized perioperative management, providing robust evidence on the clinical impact of surgical approach on pain control and recovery. In addition, the present work incorporates patient-centered outcomes—including duration of analgesic use, activity limitation, and satisfaction scores—offering a more comprehensive evaluation of postoperative quality of life. The study also quantitatively links shorter operative time in the transcanal group with faster recovery, supporting the principles of enhanced recovery after surgery (ERAS) and outpatient otologic care. To our knowledge, this is the first 14-day comparative analysis that systematically integrates surgical, analgesic, and functional parameters between transcanal and retroauricular tympanoplasty in adults.

## 5. Conclusions

This retrospective comparative study suggested that transcanal tympanoplasty may be associated with significantly lower postoperative pain, shorter operative time, earlier cessation of analgesic use, and less interference with daily activities than retroauricular tympanoplasty. These differences may be related to the minimally invasive nature of the transcanal approach, reduced soft tissue dissection, and proximity of the graft harvest to the operative field.

As outpatient tympanoplasty is becoming increasingly common, optimizing patient comfort and accelerating recovery are paramount. Our findings indicate that transcanal techniques could represent a viable option, given their surgical efficacy and superior, patient-centered outcomes. Notably, patients in the transcanal group tended to achieve lower pain levels before POD 10 and returned to normal function more rapidly, aligning with ERAS principles.

This study provides meaningful evidence in favor of an exclusively transcanal approach in ear surgery; however, further prospective randomized controlled trials are warranted to validate these findings, explore long-term auditory outcomes, and assess its cost-effectiveness across diverse patient populations and healthcare systems.

## Figures and Tables

**Table 1:** Demographic characteristics of patients.

Variable	Group I (*n* = 24)	Group II (*n* = 21)	Effect Size/OR (95% CI) ^b^	*p*
Age at surgery, y	45.5 ± 11.3 ^a^	47.2 ± 11.6 ^a^	0.37 (−8.69–5.14)	0.439
Operation time (minutes)	55.4 ± 10.1 ^a^	90.2 ± 10.6 ^a^	9.15 (−60.00–−41.18)	<0.001 *
Gender				0.759
Male	10 (41.7%)	7 (33.3%)	1.25 (0.58–2.70)	
Female	14 (58.3%)	14 (66.7%)	0.88 (0.56–1.38)	
Perforation location				1.000
Central	21 (87.5%)	18 (85.7%)	1.02 (0.81–1.29)	
Marginal	3 (12.5%)	3 (14.3%)	0.88 (0.20–3.88)	

CI: confidence intervals. * *p* < 0.05. SPSS version 25.0 (IBM Corp., Armonk, NY, USA). ^a^ Presented as mean ± standard deviation. ^b^ For continuous variables, between-group differences are presented as absolute differences with corresponding 95% confidence intervals. For categorical variables, effect estimates are expressed as differences in proportions between groups with associated 95% confidence intervals.

## Data Availability

The datasets used and/or analyzed during the current study are available from the corresponding author on reasonable request.

## References

[1] Toulouie S., Block-Wheeler N.R., Rivero A. (2022). Postoperative Pain After Endoscopic vs Microscopic Otologic Surgery: A Systematic Review and Meta-analysis. Otolaryngol. Head. Neck Surg..

[2] Carmel Neiderman N.N., Frisch M., Handzel O. (2022). Characterization of pain after tympanoplasty and tympanomastoidectomy and analysis of risk factors. A prospective cohort study. Eur. Arch. Otorhinolaryngol..

[3] Rawal N. (2005). Organization, function, and implementation of acute pain service. Anesth. Clin. North Am..

[4] Baazil A.H.A., van Spronsen E., Ebbens F.A., Dikkers F.G., De Wolf M.J.F. (2021). Pain after ear surgery: A prospective evaluation of endoscopic and microscopic approaches. Laryngoscope.

[5] Gupta A., Kaur K., Sharma S., Goyal S., Arora S., Murthy R.S.R. (2010). Clinical aspects of acute post-operative pain management and its assessment. J. Adv. Pharm. Technol. Res..

[6] Nair G.M., Birnie D.H., Sumner G.L., Krahn A.D., Healey J.S., Nery P.B., Kalfon E., Verma A., Ayala-Paredes F., Coutu B. (2021). Post-operative pain following cardiac implantable electronic device implantation: Insights from the BRUISE CONTROL trials. Europace.

[7] Chen C.K., Hsu H.C., Wang M. (2022). Endoscopic tympanoplasty with post-conchal perichondrium in repairing large-sized eardrum perforations. Eur. Arch. Otorhinolaryngol..

[8] Ayache S., Beltran M., Guevara N. (2019). Endoscopic transcanal myringoplasty for anterior tympanic membrane perforation. Eur. Ann. Otorhinolaryngol. Head. Neck Dis..

[9] Kwinter A., Purcell P.L., Leonard C.G., James A.L. (2021). Comparing transcanal endoscopic ear surgery to post-auricular microscope-guided surgery in pediatric ossiculoplasty: Hearing outcomes and postoperative pain. Otol. Neurotol..

[10] Wong D.L., Baker C.M. (1988). Wong-Baker Faces Pain Rating Scale. PsycTESTS. https://wongbakerfaces.org/.

[11] Huskisson E.C. (1974). Measurement of pain. Lancet.

[12] Faul F., Erdfelder E., Buchner A., Lang A.G. (2009). Statistical power analyses using G*Power 3.1: Tests for correlation and regression analyses. Behav. Res. Methods.

[13] Wang T.C., Shih T.C., Chen C.K., Hsieh V.C.R., Lin D.J., Tien H.C., Chen K.C., Tsai M.H., Lin C.D., Tsai C.H. (2024). Endoscopic Versus Microscopic Type I Tympanoplasty: An Updated Systematic Review and Meta-analysis. Otolaryngol. Head. Neck Surg..

[14] Rontal M., Rontal E. (1977). Lesions of the vagus nerve: Diagnosis, treatment and rehabilitation. Laryngoscope.

[15] Li K.H., Chan L.P., Chen C.K., Kuo S.H., Wang L.F., Chang N.C., Wang H.M., Ho K.Y., Chien C.Y. (2021). Comparative study of endoscopic and microscopic type I tympanoplasty in terms of delayed facial palsy. Otolaryngol. Head. Neck Surg..

[16] Bianchini A.J., Berlitz V.G., Mocelin A.G., Ribeiro J.F., Keruk J.G., Hamerschmidt R. (2023). Endoscopic or microscopic tympanoplasty advantages and disadvantages: A theory domain systematic review. Int. Arch. Otorhinolaryngol..

[17] Chorath K., Hobday S., Suresh N.V., Go B., Moreira A., Rajasekaran K. (2022). Enhanced recovery after surgery protocols for outpatient operations in otolaryngology: Review of literature. World J. Otorhinolaryngol. Head. Neck Surg..

